# Impact of Education on Inappropriate Antibiotic Prescription for Respiratory Tract Infection Based on Physicians’ Justifications: A Web-Based Survey in Japan

**DOI:** 10.3390/antibiotics13111022

**Published:** 2024-10-30

**Authors:** Ryohei Kudoh, Kosaku Komiya, Norihito Kaku, Yuichiro Shindo, Tatsuya Hayashi, Kei Kasahara, Tomohiro Oishi, Naruhiko Ishiwada, Makoto Ito, Hiroshi Yotsuyanagi, Naoki Hasegawa, Kazuhiro Tateda, Muneki Hotomi, Katsunori Yanagihara

**Affiliations:** 1 Respiratory Medicine and Infectious Diseases, Oita University Faculty of Medicine, 1-1 Idaigaoka, Hasama-machi, Yufu, Oita 879-5593, Japan; 2 Department of Laboratory Medicine, Nagasaki University Hospital, 1-7-1 Sakamoto, Nagasaki 852-8501, Japan; 3 Department of Respiratory Medicine, Nagoya University Graduate School of Medicine, 65 Tsurumai-cho, Showa-ku, Nagoya 466-8550, Japan; 4 Department of Otolaryngology-Head and Neck Surgery, Asahikawa Medical University, Midorigaoka Higashi 2-1-1-1, Asahikawa 078-8510, Japan; 5 Department of Infectious Diseases, Nara Medical University, 840 Shijo-cho, Kashihara, Nara 634-8521, Japan; 6 Department of Clinical Infectious Diseases, Kawasaki Medical School, 577 Matsuyama, Kurashiki 701-0192, Japan; 7 Department of Infectious Diseases, Medical Mycology Research Center, Chiba University, 1-8-1 Inohana, Chuo-ku, Chiba 260-8673, Japan; 8 Department of Otolaryngology and Head and Neck Surgery, Jichi Medical University, 3311-1 Yakushiji, Shimotsuke-shi, Tochigi 329-0431, Japan; 9 Department of Infectious Diseases and Applied Immunology, IMSUT Hospital, Institute of Medical Science, University of Tokyo, 4-6-1 Shirokanedai, Minato-ku, Tokyo 108-8639, Japan; 10 Department of Infectious Diseases, Keio University School of Medicine, 35 Shinano-machi, Shinjuku-ku, Tokyo 160-8582, Japan; 11 Department of Microbiology and Infectious Disease, Toho University School of Medicine, 5-21-16 Omori-nishi, Ota-ku, Tokyo 143-8540, Japan; 12 Department of Otorhinolaryngology-Head and Neck Surgery, Wakayama Medical University, 811-1 Kimiidera, Wakayama 641-8509, Japan

**Keywords:** antibiotics, respiratory tract infection, prescription, education, drug-resistant

## Abstract

**Background**: Antibiotics are inappropriately prescribed for respiratory tract infections for various reasons. The differences of the effects of education based on these reasons has not been fully elucidated. This study assessed the impact of an educational film on antibiotic prescription patterns according to physicians’ prescribing justifications. **Methods**: This was a secondary analysis of a nationwide web-based survey involving 1100 physicians. The physicians were required to view a short educational film and determine the need for prescribing antibiotics in simulated scenarios of different acute respiratory tract infectious diseases. The associations between the reasons for antibiotic prescription to patients not requiring antibiotics before viewing the educational film and the positive effects of the intervention were analyzed. **Results**: The educational intervention positively affected prescribing trends among physicians who prescribed antibiotics for “fever” in mild acute rhinosinusitis (prescription rates from 100% to 25.9%), “pus in the laryngopharynx” in mild acute pharyngitis (prescription rates from 100% to 29.6%), and “purulent sputum” in acute bronchitis without chronic lung disease (prescription rates from 100% to 29.9%) before viewing the film. In contrast, no benefits were observed when the justification was “patient’s desire for antibiotics” in mild acute pharyngitis (prescription rates from 100% to 48.5%) and acute bronchitis without chronic lung disease (prescription rates from 100% to 44.0%) or “parents’ desire for antibiotics” in narrowly defined common cold in children (prescription rates from 100% to 45.7%). **Conclusions**: although educational interventions might reduce inappropriate antibiotic prescription by providing accurate knowledge about respiratory tract infections, they appear ineffective for physicians who prescribe antibiotics based on patients’ or parents’ desires for antibiotic treatment.

## 1. Introduction

The increasing prevalence of drug-resistant pathogens is a global concern, and improper antibiotic use needs to be urgently reduced [1]. The emergence of drug-resistant organisms is correlated to the level of antibiotic consumption, and effective and sustainable interventions to ensure proper use of antibiotics are required [2]. Respiratory tract infection (RTI) is a major disease that requires proper antibiotic usage, as antibiotics are often prescribed even though most RTIs are caused by a viral infection [3]. A meta-analysis revealed that the antimicrobial prescription rate was 25–90% [4]. Physicians provide various explanations regarding antibiotic treatment selection; for example, “fever”, “pus in the laryngopharynx”, and “purulent sputum” have been identified as clinical factors associated with antibiotic prescription for RTI [5]. However, these symptoms and findings are not accurate indicators of the need for antibiotics [6]. To reduce improper antibiotic prescription, education on the updated concept of RTIs and indications for antibiotic use for clinical physicians is crucial.

We previously conducted a nationwide web-based interventional study to determine physicians’ justifications for prescribing antibiotics in fictional scenarios and compared the prescription rates before and after viewing an original educational film on basic knowledge of RTIs and the indications for antibiotic treatment [7]. The rate of antibiotic prescription for cases not requiring antibiotic treatment was significantly decreased by viewing the educational film, and some factors, including physicians’ backgrounds, were associated with antibiotic prescription patterns. However, the reasons for antibiotic prescription in cases not requiring antibiotic treatment varied, and the association between the reasons for antibiotic prescription before viewing the educational film and positive effects of the intervention has not been analyzed. If physicians prescribe antibiotics for reasons that are unrelated to medical knowledge, then the educational intervention based on scientific evidence could be ineffective, and other interventions could be required. Therefore, this study investigated the impact of an educational film on antibiotic prescription patterns based on physicians’ prescribing justifications.

## 2. Methods

### 2.1. Study Design and Participants

This was a secondary analysis of the prospective web-based interventional study using an online network platform (PLAMED Inc., Tokyo, Japan) [7]. This platform performs severance on medical care for its members, who work as medical workers in Japan. This survey targeted generalists (n = 660), pulmonologists (n = 220), and otorhinolaryngologists (n = 220) who were registered in the online network platform in advance. This study was conducted as part of a campaign to ensure the proper use of antibiotics for RTIs in Japan conducted by the Japanese Association of Infectious Diseases (JAID) and approved by the Institutional Ethics Committee of the Oita University Faculty of Medicine (approval no. 2509, 31 March 2023). Informed consent was obtained from all participants before participating in the survey. All aspects of this study complied with the Declaration of Helsinki. This study was financially supported by an Independent Medical Education Grant from Pfizer Japan, Inc. (Tokyo, Japan).

### 2.2. Fictional Scenarios and the Educational Film

The physicians were asked to determine whether antibiotics were indicated for various fictional scenarios and to provide reasons for their decisions. The reasons were also selected from multiple choices (overlap was allowed). The study set consisted of 10 simulated scenarios, for which participants assessed the need for antibiotic treatment. In the current analyses, we focused on cases of RTIs for which antibiotic treatment was unnecessary to evaluate the effects of an educational film on proper antibiotic usage. The case types included “narrowly defined common cold in adults”, “mild acute rhinosinusitis in an adult”, “mild acute pharyngolaryngitis in an adult”, “acute bronchitis without any underlying respiratory diseases in an adult”, “narrowly defined common cold in a 2-year-old child”, and “narrowly defined common cold in a 10-year-old child”. Detailed information about the questionnaire is available in the original publication [7].

The educational film was created by JAID committee members according to international guidelines [8,9,10,11,12,13,14]. To summarize the film, if a patient had mixed symptoms, including nasal, laryngopharyngeal, and lower respiratory tract symptoms, then a diagnosis of “narrowly defined common cold” was required. Most causative pathogens are viruses, and antibiotics are not required. If a patient predominantly complained of nasal symptoms, then a diagnosis of “acute rhinosinusitis” would be rendered. Even in the case of acute bacterial rhinosinusitis, mild cases can resolve without antibiotics, and only moderate or severe acute bacterial rhinosinusitis requires antibiotics. If a patient predominantly complained of laryngopharyngeal symptoms, then the diagnosis was “acute laryngopharyngitis”. Most cases are caused by viruses; however, acute laryngopharyngitis caused by Group A *Streptococcus* requires antibiotics. Finally, if a patient predominantly complained of lower respiratory tract symptoms such as cough, then the diagnosis was “acute bronchitis”. Most cases of acute bronchitis are caused by viruses, and antibiotic treatment is not needed.

The short film (approximately 5 min) is available at https://www.youtube.com/watch?app=desktop&v=yFsTKggL4IU (English version, accessed on 1 September 2024) and https://www.youtube.com/watch?app=desktop&v=DPe4OJDFAyw (Japanese version, accessed on 1 September 2024).

### 2.3. Statistical Analysis

Statistical analyses were performed using the Statistical Package for the Social Sciences version 22 (IBM, Armonk, NY, USA). For two-tailed analyses, 95% confidence intervals were calculated. The associations between the physicians’ justifications for antibiotic prescription to cases not requiring antibiotic treatment and the effects of the educational film were analyzed by binomial logistic regression. *p* < 0.05 was considered statistically significant.

## 3. Results

The reasons for prescribing antibiotics in cases in which they were not required before viewing the educational film varied among the types of RTIs (Table 1). The most common reasons for improper antibiotic prescription were as follows: “redness of the pharynx”, “patient’s desire”, “fever”, and “sore throat” in narrowly defined common cold; “patient’s desire” and “fever” in mild acute rhinosinusitis; “white lesion of pharynx”, “fever”, “sore throat”, and “patient’s desire” in mild acute pharyngitis; “purulent sputum”, “fever”, and “patient’s desire” in acute bronchitis without chronic lung disease; “parent’s desire”, “fever”, “nasal discharge”, and “cough” in narrowly defined common cold in a 2-year-old child; and “parent’s desire”, “fever”, “cough”, and “nasal discharge” in narrowly defined common cold in a 10-year-old child. The analyses based on physician’s specialty did not exhibit any specific trends (Supplementary Tables S1–S3).

**Figure 1:**
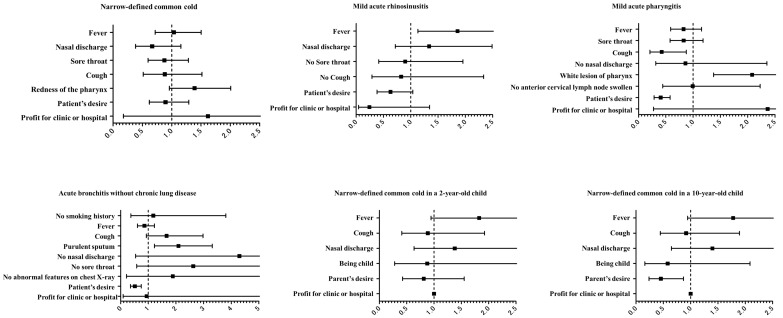
Odds ratios with 95% confidence interval for not prescribing antibiotics in cases not requiring antibiotic treatment after viewing the educational film.

The proportion of physicians who selected “fever” as the reason for antibiotic prescription in cases of mild acute rhinosinusitis significantly decreased from 100% before viewing the educational film to 25.9% after viewing the film. They were more likely to refrain from prescribing antibiotics after viewing the film than physicians who did not select “fever” in cases of mild acute rhinosinusitis before viewing the film (odds ratio [OR] = 1.857, *p* = 0.014, Figure 1). Similar trends were observed among physicians who selected “white lesion of pharynx” in cases of mild acute pharyngitis (prescription rate decreased from 100% to 29.6%), and the change was significant compared with that in doctors who did not select this indication (OR = 2.025, *p* = 0.014). In addition, the proportion of physicians who prescribed antibiotics for “purulent sputum” in acute bronchitis without chronic lung disease decreased from 100% to 29.9% after viewing the film, and these physicians were more likely to refrain from prescribing antibiotics than their counterparts who did not select this indication (OR = 2.089, *p* = 0.001). The physician’s specialty did not affect these trends (Supplementary Figures S1–S3).

Conversely, although physicians who prescribed antibiotics because of “patient’s desire” in cases of mild acute pharyngitis were less likely to prescribe antibiotics after viewing the film (the prescription rate decreased from 100% to 48.5%), they were less likely to refrain from prescribing antibiotics after viewing the film than physicians who did not select “patient’s desire” as the justification (OR = 0.406, *p* < 0.001). Similarly, the educational film had little effect on physicians who selected “cough” in cases of mild acute pharyngitis (decrease from 100% to 51.6%), although they remained significantly less likely to prescribe antibiotics after the intervention than their counterparts who did not select this justification (OR = 0.425, *p* = 0.017). Meanwhile, the proportion of physicians who prescribed antibiotics because of “patient’s desire” in cases of acute bronchitis without chronic lung disease decreased from 100% before the intervention to 44.0% after the intervention, and they were less likely to prescribe antibiotics after viewing the film than physicians who did not select this indication (OR = 0.510, *p* < 0.001). The proportion of physicians who prescribed antibiotics because of “parent’s desire” in narrowly defined common cold in a 10-year-old child fell from 100% to 45.7%, and they were less likely to prescribe antibiotics after viewing the film than physicians who did not select this indication (OR = 0.455, *p* = 0.016).

## 4. Discussion

The current study demonstrated that educational training targeting proper antibiotic use substantially reduced unnecessary antibiotic prescriptions among physicians who prescribed antibiotic treatment because of “fever” in cases of mild acute rhinosinusitis, “white lesion of pharynx” in cases of mild acute pharyngitis, and “purulent sputum” in cases of acute bronchitis without chronic lung disease in adults. These results are consistent with those of a systematic review that evaluated factors associated with antibiotic prescribing patterns in patients with RTIs [5]. The meta-analysis in this review revealed that antibiotic prescription had significant positive relationships with physical findings such as “fever”, “tonsillar exudate”, and “purulent sputum”. Consequently, if inappropriate antibiotic prescribing is attributable to incorrect knowledge or a lack of knowledge, the educational interventions for physicians could be effective. Conversely, physicians who prescribed antibiotic treatment at the request of patients or their families had significantly lower reduction rates after viewing the educational film than their counterparts. Patients’ or parents’ desires have been reported to be related to antibiotic prescription trends [15,16]. However, the current study revealed that the educational intervention was ineffective for physicians who prescribed antibiotics for these reasons. One possible explanation is that physicians’ concerns about patients’ satisfaction and the popularity of the clinic or hospital influenced their prescribing decisions. Indeed, patients hoping for antibiotics were less satisfied when antibiotics were not prescribed, and patients’ desires or clinicians’ perceptions that patients want to be prescribed antibiotics have been linked to antibiotic prescribing trends [17]. Furthermore, another study reported that antibiotics might be prescribed by physicians to help maintain a good relationship with patients [18]. In the current study, “profit for clinic or hospital” as a reason for antibiotic prescription was not associated with a change in the antibiotic treatment pattern. This justification was not influenced by the educational intervention for physicians. Presumably, physicians who are simply concerned about patients’ satisfaction or hoped to maintain good relationships were not willing to absorb the knowledge regarding the proper use of antibiotics for RTIs. Meanwhile, physicians who prescribed antibiotics for “cough” in cases of mild acute pharyngitis also displayed a lower rate of reduction of antibiotic prescription after the intervention. In the educational film, cough was not mentioned in the section on acute pharyngitis, which might have weakened the educational effect in this case.

Considering the lack of benefit for the intervention among physicians who prescribed antibiotics based on patients’ or parents’ desires, other interventions should be considered. First, educational approaches for patients or their families could be successful. One study found that posters targeting patients were potentially useful for increasing appropriate antibiotics prescribing. Meeker et al. conducted a randomized clinical trial in the US, revealing that posting a notice regarding the proper use of antibiotics in the examination room resulted in a 19.7% reduction in inappropriate antibiotic prescribing [19]. They claimed that this simple intervention could eliminate 2.6 million unnecessary antibiotic prescriptions and save USD 70.4 million annually on drug costs. In addition, public education could also be successful. An investigation in Laos noted that public educational activities improved awareness and understanding regarding antimicrobial resistance [20]. The average recognition of “drug resistance” rose from 27.6% to 91.4% among the participants, compared with an increase from 36.2% to 58.8% in the unexposed group. Based on this evidence, educational interventions for both physicians and the general public appear necessary. Furthermore, intervention as a form of reimbursement can be considered. In fact, an additional fee for appropriate antibiotic use among children was introduced in Japan in 2018. This program significantly reduced antibiotic prescribing rates by almost 18% without consequent increases in hospitalization rates or after-hours visits [21].

The strength of the current study was its use of a relatively large nationwide survey focusing on the effects of an educational intervention on inappropriate antibiotic prescription trends for RTIs based on physicians’ justifications. Although previous studies have revealed the factors associated with antibiotic prescription, no study has focused on the relationship between an educational intervention and physicians’ justifications. Our study has some limitations that must be addressed. First, this survey assessed the changes in antibiotic prescription patterns immediately after viewing a short educational film. Although the educational intervention exhibited short-term efficacy, the long-lasting effects remain uncertain. Second, the fictional scenarios could not cover all situations in which patients visit clinics or hospitals for RTIs in clinical practice. Although the scenarios covered a variety of RTI types, the number of cases was limited for this survey. Finally, this study was conducted in Japan, which has universal health coverage. Thus, caution must be exercised when generalizing the results of the current survey considering each country or region’s medical care system.

In conclusion, this study revealed that an educational film effectively reduced inappropriate antibiotic prescription associated with misinformation or a lack of knowledge among physicians. However, the intervention did not reduce prescribing rates associated with patients’ or parents’ desires. Interventions to educate physicians and the general public or medical reimbursement could be necessary.

## Figures and Tables

**Table 1:** Reasons for antibiotic prescription in cases not requiring antibiotic treatment before viewing the educational film.

**Narrowly Defined Common Cold** **(n = 557)**		**Mild Acute Rhinosinusitis** **(n = 300)**		**Mild Acute Pharyngitis** **(n = 775)**	
Fever	251 (45.1%)	Fever	135 (45.0%)	Fever	208 (26.8%)
Nasal discharge	63 (11.3%)	Nasal discharge	60 (20.0%)	Sore throat	170 (21.9%)
Sore throat	190 (34.1%)	No sore throat	31 (10.3%)	Cough	31 (4.0%)
Cough	71 (12.7%)	No cough	16 (5.3%)	No nasal discharge	17 (2.2%)
Redness of the pharynx	304 (54.6%)	Patient’s desire	176 (58.7%)	White lesion of pharynx	668 (86.2%)
Patient’s desire	264 (47.4%)	Profit for clinic or hospital	6 (2.0%)	No anterior cervical lymph node swollen	28 (3.6%)
Profit for clinic or hospital	5 (0.9%)			Patient’s desire	163 (21.0%)
				Profit for clinic or hospital	6 (0.8%)
**Acute Bronchitis Without Chronic Lung Disease** **(n = 688)**		**Narrowly Defined Common Cold** **in a 2-Year-Old Child** **(n = 153)**		**Narrowly Defined Common Cold** **in a 10-Year-Old Child** **(n = 168)**	
No smoking history	14 (2.0%)	Fever	66 (43.1%)	Fever	87 (51.8%)
Fever	198 (28.8%)	Cough	33 (21.6%)	Cough	41 (24.4%)
Cough	70 (10.2%)	Nasal discharge	35 (22.9%)	Nasal discharge	38 (22.6%)
Purulent sputum	603 (87.6%)	Being child	13 (8.5%)	Being child	10 (6.0%)
No nasal discharge	10 (1.5%)	Parent’s desire	86 (56.2%)	Parent’s desire	92 (54.8%)
No sore throat	13 (1.9%)	Profit for clinic or hospital	2 (1.3%)	Profit for clinic or hospital	0 (0.0%)
No abnormal features on chest X-ray	5 (0.7%)				
Patient’s desire	150 (21.8%)				
Profit for clinic or hospital	3 (0.4%)				

Data are presented as n (%).

## Data Availability

The original contributions presented in this study are included in the article and Supplementary Materials, further inquiries can be directed to the corresponding author.

## Supplementary Materials

The following supporting information can be downloaded at: https://www.mdpi.com/article/10.3390/antibiotics13111022/s1. Table S1. Reasons for antibiotics prescription in generalists before viewing the educational films in cases not requiring antibiotic treatment. Table S2. Reasons for antibiotics prescription in pulmonologists before viewing the educational films in cases not requiring antibiotic treatment. Table S3. Reasons for antibiotics prescription in otorhinolaryngologists before viewing the educational films in cases not requiring antibiotic treatment. Figure S1. Odds ratios with 95% confidence interval of not prescribing in generalists after viewing the films in cases not requiring antibiotic treatment. Figure S2. Odds ratios with 95% confidence interval of not prescribing in pulmonologists after viewing the films in cases not requiring antibiotic treatment. Figure S3. Odds ratios with 95% confidence interval of not prescribing in otorhinolaryngologists after viewing the films in cases not requiring antibiotic treatment.
