# Supplementary material for: Impact of Education on Inappropriate Antibiotic Prescription for Respiratory Tract Infection Based on Physicians’ Justifications: A Web-Based Survey in Japan
Source: Antibiotics (Basel). 2024 Oct 30;13(11):1022. doi: 10.3390/antibiotics13111022 (PMC11591160; doi:10.3390/antibiotics13111022)
Supplement: Supplementary file 1 [file antibiotics-13-01022-s001.zip › antibiotics-3255573-supplementary.pdf]

Table S1. Reasons for antibiotics prescription in generalists before viewing the educational films in cases not requiring antibiotic treatment.

Case of narrow-defined common cold (n = 330)		Case of mild acute rhinosinusitis (n = 181)		Case of mild acute pharyngitis (n = 436)	
Fever	148 (44.8%)	Fever	79 (43.6%)	Fever	114 (26.1%)
Nasal discharge	31 (9.4%)	Nasal discharge	31 (17.1%)	Sore throat	93 (21.3%)
Sore throat	103 (31.2%)	No sore throat	19 (10.5%)	Cough	20 (4.6%)
Cough	43 (13.0%)	No cough	10 (5.5%)	No nasal discharge	13 (2.0%)
Redness of the pharynx	184 (55.8%)	Patient's desire	111 (61.3%)	White lesion of pharynx	374 (85.8%)
Patient's desire	171 (51.8%)	Profit for clinic or hospital	2 (1.1%)	No anterior cervical lymph node swollen	14 (3.2%)
Profit for clinic or hospital	1 (0.3%)			Patient's desire	100 (22.9%)
				Profit for clinic or hospital	1 (0.2%)

Case of acute bronchitis without chronic lung disease (n = 416)		Case of narrow-defined common cold in a 2-year-old child (n = 81)		Case of narrow-defined common cold in a 10-year-old child (n = 91)	
No smoking history	10 (2.4%)	Fever	33 (40.7%)	Fever	41 (45.1%)
Fever	113 (27.2%)	Cough	20 (24.7%)	Cough	23 (25.3%)
Cough	43 (10.3%)	Nasal discharge	14 (17.3%)	Nasal discharge	15 (16.5%)
Purulent sputum	360 (86.5%)	Being child	4 (4.9%)	Being child	4 (4.4%)
No nasal discharge	5 (1.2%)	Parent's desire	55 (67.9%)	Parent's desire	58 (63.7%)
No sore throat	9 (2.2%)	Profit for clinic or hospital	0 (0%)	Profit for clinic or hospital	0 (0.0%)
No abnormal features on chest X-ray	4 (1.0%)				
Patient's desire	92 (22.1%)				
Profit for clinic or hospital	1 (0.2%)				

Data are presented as the number (%)

Table S2. Reasons for antibiotics prescription in pulmonologists before viewing the educational films in cases not requiring antibiotic treatment.

Case of narrow-defined common cold (n = 98)		Case of mild acute rhinosinusitis (n = 58)		Case of mild acute pharyngitis (n = 162)	
Fever	48 (49.0%)	Fever	30 (51.7%)	Fever	50 (30.9%)
Nasal discharge	12 (12.2%)	Nasal discharge	12 (20.7%)	Sore throat	32 (19.8%)
Sore throat	32 (32.7%)	No sore throat	7 (12.1%)	Cough	7 (4.3%)
Cough	17 (17.3%)	No cough	5 (8.6%)	No nasal discharge	2 (1.2%)
Redness of the pharynx	49 (50.0%)	Patient's desire	34 (58.6%)	White lesion of pharynx	143 (88.3%)
Patient's desire	51 (52.0%)	Profit for clinic or hospital	1 (1.7%)	No anterior cervical lymph node swollen	7 (4.3%)
Profit for clinic or hospital	3 (3.1%)			Patient's desire	33 (20.4%)
				Profit for clinic or hospital	2 (1.2%)

Case of acute bronchitis without chronic lung disease (n = 142)		Case of narrow-defined common cold in a 2-year-old child (n = 16)		Case of narrow-defined common cold in a 10-year-old child (n = 20)	
No smoking history	2 (1.4%)	Fever	6 (37.5%)	Fever	12 (60.0%)
Fever	45 (31.7%)	Cough	4 (25.0%)	Cough	7 (35.0%)
Cough	9 (6.3%)	Nasal discharge	3 (18.8%)	Nasal discharge	2 (10.0%)
Purulent sputum	124 (87.3%)	Being child	2 (12.5%)	Being child	2 (10.0%)
No nasal discharge	4 (2.8%)	Parent's desire	8 (50.0%)	Parent's desire	12 (60.0%)
No sore throat	3 (2.1%)	Profit for clinic or hospital	1 (6.3%)	Profit for clinic or hospital	0 (0.0%)
No abnormal features on chest X-ray	1 (0.7%)				
Patient's desire	34 (23.9%)				
Profit for clinic or hospital	1 (0.7%)				

Data are presented as the number (%)

Table S3. Reasons for antibiotics prescription in otorhinolaryngologists before viewing the educational films in cases not requiring antibiotic treatment.

Case of narrow-defined common cold (n = 129)	
Fever	55 (42.6%)
Nasal discharge	20 (15.5%)
Sore throat	55 (42.6%)
Cough	11 (8.5%)
Redness of the pharynx	71 (55.0%)
Patient's desire	42 (32.6%)
Profit for clinic or hospital	1 (0.8%)

Case of mild acute rhinosinusitis (n = 61)	
Fever	26 (42.6%)
Nasal discharge	17 (27.9%)
No sore throat	5 (8.2%)
No cough	1 (1.6%)
Patient's desire	31 (50.8%)
Profit for clinic or hospital	3 (4.9%)

Case of mild acute pharyngitis (n = 177)	
Fever	44 (24.9%)
Sore throat	45 (25.4%)
Cough	4 (2.3%)
No nasal discharge	2 (1.1%)
White lesion of pharynx	151 (85.3%)
No anterior cervical lymph node swollen	7 (4.0%)
Patient's desire	30 (16.9%)
Profit for clinic or hospital	3 (1.7%)

Case of acute bronchitis without chronic lung disease (n = 130)	
No smoking history	2 (1.5%)
Fever	40 (30.8%)
Cough	18 (13.8%)
Purulent sputum	119 (91.5%)
No nasal discharge	1 (0.8%)
No sore throat	1 (0.8%)
No abnormal features on chest X-ray	0 (0%)
Patient's desire	24 (18.5%)
Profit for clinic or hospital	1 (0.8%)

Case of narrow-defined common cold in a 2-year-old child (n = 56)	
Fever	27 (48.2%)
Cough	9 (16.1%)
Nasal discharge	18 (32.1%)
Being child	7 (12.5%)
Parent's desire	23 (41.1%)
Profit for clinic or hospital	1 (1.8%)

Case of narrow-defined common cold in a 10-year-old child (n = 57)	
Fever	34 (59.6%)
Cough	11 (19.3%)
Nasal discharge	21 (36.8%)
Being child	4 (7.0%)
Parent's desire	22 (38.6%)
Profit for clinic or hospital	0 (0.0%)

Data are presented as the number (%)

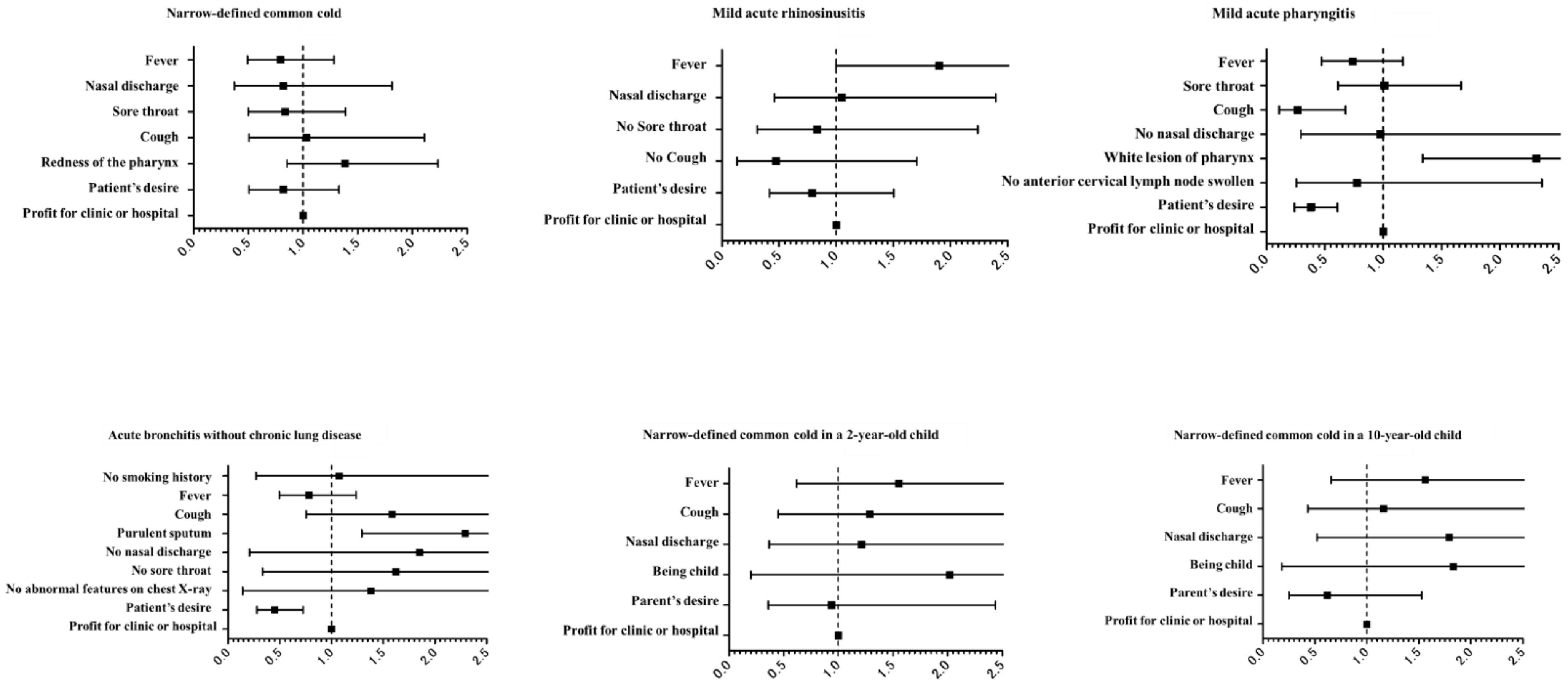

Figure S1. Odds ratios with 95% confidence interval of not prescribing in generalists after viewing the films in cases not requiring antibiotic treatment.

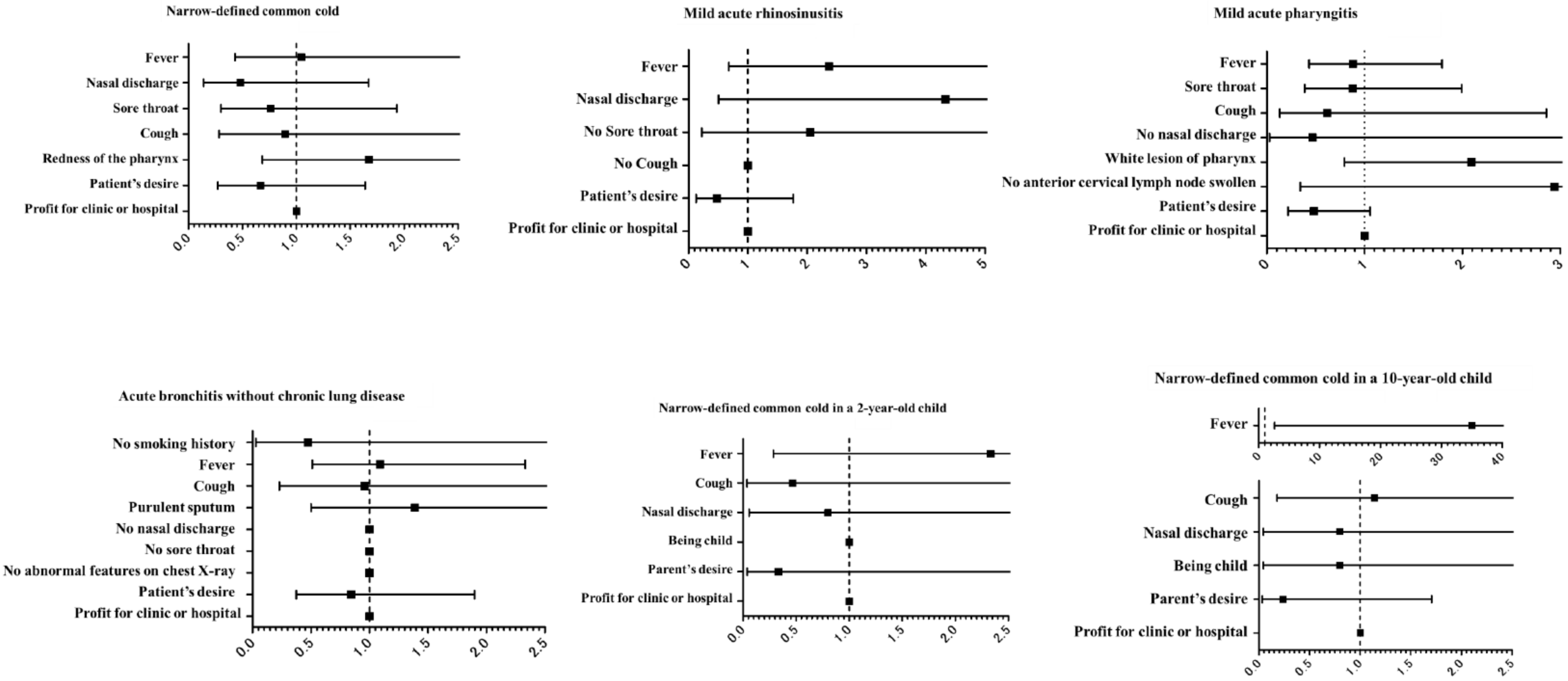

Figure S2. Odds ratios with 95% confidence interval of not prescribing in pulmonologists after viewing the films in cases not requiring antibiotic treatment.

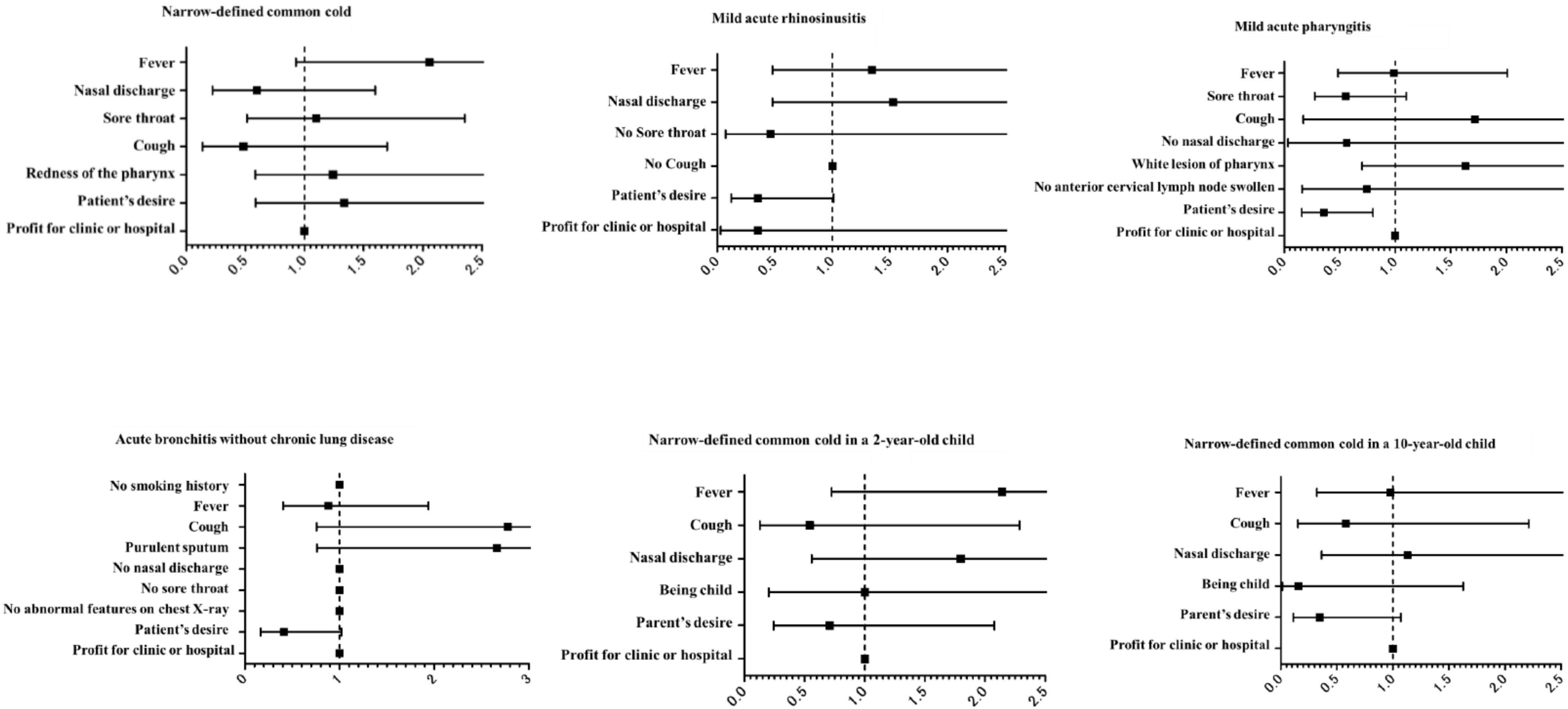

Figure S3. Odds ratios with 95% confidence interval of not prescribing in otorhinolaryngologists after viewing the films in cases not requiring antibiotic treatment.
